# Supplementary material for: SPON1 Can Reduce Amyloid Beta and Reverse Cognitive Impairment and Memory Dysfunction in Alzheimer’s Disease Mouse Model
Source: Cells. 2020 May 21;9(5):1275. doi: 10.3390/cells9051275 (PMC7290723; doi:10.3390/cells9051275)
Supplement: Supplementary file 1 [file cells-09-01275-s001.pdf]

Supplementary Materials

SPON1 can Reduce Amyloid Beta and Reverse Cognitive Impairment and Memory Dysfunction in Alzheimer's Disease Mouse Model.

Soo Yong Park [1], Joo Yeong Kang [1], Taehee Lee [1], Donggyu Nam [1], Chang-Jin Jeon [2] and Jeong Beom Kim [1,*]

[1] Stem Cell Research Center, School of Life Sciences, Ulsan National Institute of Science and Technology (UNIST), 44919 Ulsan, South Korea; shorypark@unist.ac.kr (S.Y.P.); kangj0594@unist.ac.kr (J.Y.K.); thlee525@hotmail.com (T.L.); namdongg@unist.ac.kr (D.N.)

[2] Neuroscience Laboratory, Department of Biology, College of Natural Sciences, Kyungpook National University, 41566, Daegu, South Korea; cjjeon@knu.ac.kr

* Correspondence: jbkim@unist.ac.kr; Tel: +82 (52) 217 5272

Table S1. Sequence information used in this study.	
**name**	sequence
exo-Spon1 forward	5′-CCAAACTGTGCGGAGGTGG-3′
exo-Spon1 reverse	5′-AGGTAGCGGTGAGTTCAGGC-3′
exo-Reelin/spondin forward	5′-GGGATGCTGGCACTGACAG-3′
exo-Reelin/spondin reverse	5′-AGGTAGCGGTGAGTTCAGGC-3′
endo-Spon1 forward	5′-CGTCACTTGGAGCAGGAGTG-3′
endo-Spon1 reverse	5′-CCCTCCGACTTGGTCACTTT-3′
Gapdh qRT forward	5′-TGCCCCCATGTTTGTGAT-3′
Gapdh qRT reverse	5′- TGTGGTCATGAGCCCTTC-3′
Sox2 forward	5′-TAGAGCTAGACTCCGGGCGATGA-3′
Sox2 reverse	5′-TTGCCTTAAACAAGACCACGAAA-3′
Nestin forward	5′-TTGACCTGTGGTATCTCTCGTG-3′
Nestin reverse	5′-GAGTCACTCATCATTGCTGCTC-3′
Pax6 forward	5′-GCTTCATCCGAGTCTTCTCCGTTAG-3′
Pax6 reverse	5′-CCATCTTTGCTTGGGAAATCCG-3′
Olig2 forward	5′-CAGAGCACTGCACTTGACTTCT-3′
Olig2 reverse	5′-CACACTTCTGAGACCACAGGAC-3′
Gapdh forward	5′-ACGACCCCTTCATTGACCTCAACT-3′
Gapdh reverse	5′-ATATTTCTCGTGGTTCACACCCAT-3′

Table S2. Primary antibodies used in this study

**Antibody**	**Application**	**Host species**	**Company**	Catalog number
Nestin	1:400 (ICC)	Mouse	Millipore	MAB353
Sox2	1:200 (ICC)	Goat	SantaCruz	sc-17320
V5	1:200 (ICC) 1:1000 (WB)	Goat	abcam	ab95038
Aβ	1:1000 (WB)	Mouse	Novus	NBP2-13075
HSC-70	1:1000 (WB)	Rabbit	abcam	ab137806

*ICC: immunocytochemistry, WB: western blot

Table S3. Morris water maze mouse start positions

**Day**	**Trial 1**	**Trial 2**	**Trial 3**	Trial 4
1	N	E	SE	NW
2	SE	N	NW	E
3	NW	SE	E	N
4	E	NW	N	SE
5	N	SE	E	NW
6 (probe trial)	NE			

N: North, E: East, SE: South East, NW: North West, NE: North East.

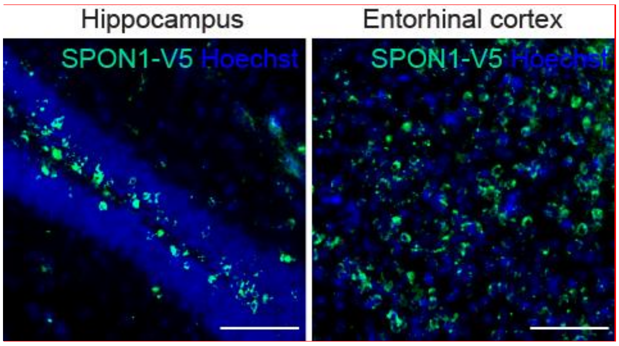

Figure S1. Immunofluorescence images of ectopic expression of human SPON1 in injected sites hippocampus (left panel) and entorhinal cortex (right panel). Anti-V5 antibody was used to detect V5-tagged SPON1. Scale bar: 100 μm.
